# Large-Area Nanopillar Arrays by Glancing Angle Deposition with Tailored Magnetic Properties

**DOI:** 10.3390/nano12071186

**Published:** 2022-04-01

**Authors:** Elena Navarro, María Ujué González, Fanny Béron, Felipe Tejo, Juan Escrig, José Miguel García-Martín

**Affiliations:** 1 Instituto de Magnetismo Aplicado, Universidad Complutense de Madrid-ADIF-CSIC, P.O. Box 155, Las Rozas, 28230 Madrid, Spain; 2 Departamento de Física de Materiales, Universidad Complutense de Madrid, 28040 Madrid, Spain; 3 Instituto de Micro y Nanotecnología, IMN-CNM, CSIC (CEI UAM+CSIC), Isaac Newton 8, Tres Cantos, 28760 Madrid, Spain; maria-ujue.gonzalez@csic.es (M.U.G.); josemiguel.garcia.martin@csic.es (J.M.G.-M.); 4 Instituto de Física Gleb Wataghin, Universidade Estadual de Campinas (UNICAMP), Campinas 13083-859, SP, Brazil; fberon@ifi.unicamp.br; 5 Departamento de Física, Facultad de Ciencia, Universidad de Santiago de Chile (USACH), Santiago 9170124, Chile; felipe.tejo@icmm.csic.es (F.T.); juan.escrig@usach.cl (J.E.); 6 Instituto de Ciencia de Materiales de Madrid, Cantoblanco, 28049 Madrid, Spain; 7 Center for the Development of Nanoscience and Nanotechnology (CEDENNA), Santiago 9170124, Chile

**Keywords:** glancing angle deposition, magnetron sputtering, large area nanopillars coverage, nanostructured magnetic films, anisotropic surface morphology, growth-induced magnetic anisotropy

## Abstract

Ferromagnetic films down to thicknesses of tens of nanometers and composed by polycrystalline Fe and Fe_2_O_3_ nanopillars are grown in large areas by glancing angle deposition with magnetron sputtering (MS-GLAD). The morphological features of these films strongly depend on the growth conditions. Vertical or tilted nanopillars have been fabricated depending on whether the substrate is kept rotating azimuthally during deposition or not, respectively. The magnetic properties of these nanopillars films, such as hysteresis loops squareness, adjustable switching fields, magnetic anisotropy and coercivity, can be tuned with the specific morphology. In particular, the growth performed through a collimator mask mounted onto a not rotating azimuthally substrate produces almost isolated well-defined tilted nanopillars that exhibit a magnetic hardening. The first-order reversal curves diagrams and micromagnetic simulations revealed that a growth-induced uniaxial anisotropy, associated with an anisotropic surface morphology produced by the glancing angle deposition in the direction perpendicular to the atomic flux, plays an important role in the observed magnetic signatures. These results demonstrate the potential of the MS-GLAD method to fabricate nanostructured films in large area with tailored structural and magnetic properties for technological applications.

## 1. Introduction

Glancing Angle Deposition (GLAD) is an easy and versatile route to fabricate arrays of nanostructures in large areas (cm^2^ and above) in a single processing step, in clear contrast to other techniques in the nanoscale, such as e-beam lithography and ion-beam lithography. Through a physical vapor deposition method, namely thermal evaporation, electron beam bombardment, or magnetron sputtering, a flux of particles or atoms is produced in direction of a substrate tilted by an angle of at least 70°, with this angle being measured between the normal to the substrate and the deposition flux direction. As a result of the shadowing effect provided by the nuclei formed on the substrate to the incoming flux, tilted nanopillars can be obtained [1]. Moreover, with a substrate motion, either changing the tilt angle, either rotating it around its normal (azimuthal rotation), other nanostructures can be achieved, such as vertical nanopillars, helices, and zig-zag entities [1,2,3]. Those nanostructures can be used to develop applications in areas such as optics, energy, catalysis, sensors, and biomaterials [4,5,6].

From the different physical vapor deposition techniques than can be adapted to GLAD configuration, magnetron sputtering is the adequate choice considering the effective cost, the compatibility with standard microfabrication processes, and the scalability for mass production. These are crucial aspects for an actual device using nanostructures arrays fabricated by GLAD being introduced in the market. In fact, two different works have recently reported the use of glancing angle deposition with magnetron sputtering (MS-GLAD) to cover the surface of real implants with antibacterial nanostructured coatings [7,8].

In the case of magnetic materials, although oblique deposition had already been used in the past to control the magnetic anisotropy of thin films [9,10,11,12,13], the GLAD configuration (i.e., tilt angle beyond 70°) was firstly serving to produce nanostructure arrays at the end of the 20th century [14,15]. Since then, different properties of the magnetic films obtained by GLAD have been studied: the magnetic domain structure [16,17,18,19], the magnetic anisotropy [20,21,22,23], the magneto-resistance [24,25,26], the optical and magneto-optical behaviors [27,28], and even their possible use for information storage [29,30,31]. Nevertheless, only a few of those studies refer to arrays fabricated by MS-GLAD [19,25,31,32,33]. The specific deposition method used is important not only for future application development, as indicated above, but mainly because the deposition regime differs. Two important features should be considered here: (i) the kinetic energy of the deposited particles in evaporation is of the order of 0.1 eV, whilst it reaches up to few tens eV in magnetron sputtering [34]; (ii) in evaporation all the atoms are ballistic, whereas in magnetron sputtering some atoms do not keep their original directionality after suffering collisions with plasma species [35]. These differences have a significant influence on the morphology of the obtained nanostructures and consequently on their properties. In this work, we report a systematic study of different arrays of magnetic nanostructures obtained by MS-GLAD: consisting of vertical nanopillars (NPs) fabricated with azimuthal rotation of the substrate during growth; or formed by slanted ones obtained without rotation. Moreover, we also study the influence of the optional use of a mask acting as an additional particle collimator that increases the ratio of ballistic atoms, on the array morphology and magnetic properties.

## 2. Methods

### 2.1. Synthesis

**Figure 1:**
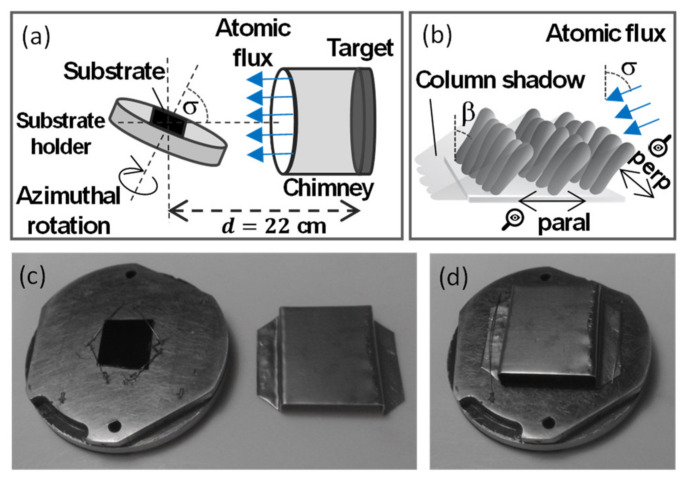
(**a**) Scheme of the sputtering process with glancing angle deposition and possible azimuthal substrate rotation. In this work, the tilt angle, being measured between the sample surface normal and the magnetron source normal, is σ=85°. (**b**) Schematic picture of the obtained nanopillars (NPs) with the definition of the directions parallel and perpendicular to the atomic flux. (**c**) Photograph of the sample mounted onto the sample holder and the mask beside them. (**d**) Photograph with the mask placed over the sample, as was used during the fabrication of two of the four samples.

Fe nanopillars (NPs) were grown by GLAD with magnetron sputtering at room temperature. We used a magnetron source from AJA (AJA Inc., Scituate, MA, USA) with a 5 cm diameter Fe target and a 22 cm distance between the target and the substrate center. Figure 1a shows a schematic diagram of the MS-GLAD setup. This method allows to produce nanostructures over large-area substrates and is based on the competition between atomic shadowing mechanisms and surface diffusion. In the ideal scenario where all the evaporated atoms are almost parallel, the atomic flux travels at the glancing angle to the substrate, where the deposited atoms nucleate forming individual separated islands. However, the regions behind those islands do not receive any further atoms because of the shadowing occurring from each nucleus. As the nuclei grow, more incoming atoms will deposit on them if the mobility of the metal atoms on the substrate is low. Otherwise, metal atoms would diffuse into the shadowed areas. This self-reinforcing behavior develops into porous films made of columnar nanostructures (see Figure 1b).

Such ideal shadowing is only possible if the incoming atomic flux is well collimated. In the case of sputtering, this means that ballistic atoms must substantially outnumber the thermalized ones [35]. Ballistic atoms are those that preserve their directionality when they travel from the source to the substrate, thus keeping their original momentum and energy when deposited onto the substrate. By contrast, thermalized species are those experiencing a large number of collisions in the plasma gas, thus possessing an isotropic momentum distribution. In order to promote the ballistic regime, in the present work we selected the minimum argon pressure necessary to maintain a stable plasma in the sputtering chamber. A cylindrical metallic chimney with 5 cm diameter and 9 cm length was also placed above the target to collimate the ballistic outgoing material flux and trap a large amount of the sputtered species that remain thermalized in the plasma phase [36]. Finally, physical screens, like the mask shown in Figure 1c,d, inhibit the deposition of low-energy atoms that may arrive at the substrate from random directions, consequently selecting a subset of the incoming atomic flux. In other words, the mask acts as an additional particle collimator [37,38]. We used a mask made of molybdenum with the following dimensions: 4 mm height, 15 mm width, and 19 mm length; thus, covering the whole substrate area.

In this work, four samples, grown in different conditions, have been analyzed. For all of them, a Si(100) substrate of about 1 cm^2^ area was used and tilted at an angle σ=85°, with σ being measured between the sample surface normal and the magnetron source normal (see Figure 1a). The base pressure of the UHV chamber was in the mid 10^−9^ mbar range. During growth of all considered samples, argon pressure, power, and deposition time were maintained at 2 × 10^−3^ mbar, 150 W, and 100 min, respectively.

The four samples analyzed differ from each other depending on whether the substrate is rotated azimuthally during the GLAD deposition at σ=85° or not, and whether the collimation mask shown in Figure 1c is or not mounted over the substrate as in Figure 1d. GLAD performed with substrate rotation gives rise to vertical nanopillars. The identification and particular growth conditions of each sample are listed in Table 1 and it is as follows: NR sample: No azimuthal rotation of the substrate during growth; NR-mask sample: No azimuthal rotation of the substrate during growth and use of mask with the atomic flux coming from one side only; R sample: Azimuthal rotation of the substrate at 3 rpm; R-mask sample: Azimuthal rotation of the substrate by 180° every 120 s (50 repetitions) so that the atomic flux enters alternatively through the two openings of the mask. For comparison purposes, a continuous thin film was also fabricated using the standard configuration with substrate parallel to the target, i.e., σ=0°, and with the same Ar pressure, power, and deposition time.

### 2.2. Characterization

The structural morphology and thickness of the deposited films were examined by field-emission scanning electron microscopy (FESEM) using a FEI Verios 460 microscope (FEI Europe B.V., Eindhoven, Netherlands). The images were obtained using low voltage, in particular 2 kV, in order to get a detailed surface structure. For each sample, top-view and cross-section images were recorded. Cross-sectional images were obtained along two different directions, perpendicular and parallel to the atomic flux, as defined in Figure 1b, by cutting the Si substrate along the desired direction. In the perpendicular configuration, the beam does not see any possible tilting of the microstructural features, while in the parallel one the observed plane contains the tilting angle of the microstructural features. The NPs crystallinity was analyzed by X-ray diffraction (XRD, Malvern PANalytical, Almelo (Netherlands) using Cu Kα (wavelength 1.5418 Å) radiation. Two PANalytical X-ray diffractometers were employed, a X’Pert MPD model in Bragg-Brentano configuration and a X’Pert MRD model in grazing incidence configuration at 0.5° with the substrate plane. Magnetic characterization was carried out by SQUID magnetometer (Quantum Design MPMS-XL, San Diego, CA, USA) while First-Order Reversal Curves (FORC) measurements were acquired in a Physical Property Measurement System from Quantum Design (PPMS-14T, San Diego, CA, USA), both at room temperature. Between 100 and 200 curves were used for the FORC distribution calculations.

### 2.3. Micromagnetic Simulations

To understand the magnetic properties and deepen into the magnetization reversal mechanism of these nanostructures, we have compared the experimental results with micromagnetic simulations using the finite difference code OOMMF. In the micro-magnetic framework, the magnetization dynamics is described using the Landau-Lifshitz-Gilbert (LLG) equation:

(1) $$\dot{m}=-\gamma \;m\times H_{eff}+\alpha \;m\times \dot{m}$$

where $m=M/M_{s}$ is the normalized magnetization, γ is the electron gyromagnetic ratio, α is the dimensionless damping parameter, and:

(2) $$H_{eff}=-\frac{1}{\mu_{0}M_{s}}\frac{\delta U\left[m\right]}{\delta m}$$

is the effective field. In the definition of $H_{eff}$, $\mu_{0}$ is the vacuum permeability, $M_{s}$ is the saturation magnetization, and $U\left[m\right]$ is the free energy functional, which considers all the energy contributions of the system. For this study, we have chosen the magnetic parameters corresponding to iron (Fe), i.e., $M_{s}=1714\;\mathrm{emu}/{\mathrm{cm}}^{3}$ and exchange constant A=21 pJ/m. We have used α=0.5 for all simulations to preserve their quasi-static regime.

## 3. Results

### 3.1. Structural and Morphological Results

**Figure 2:**
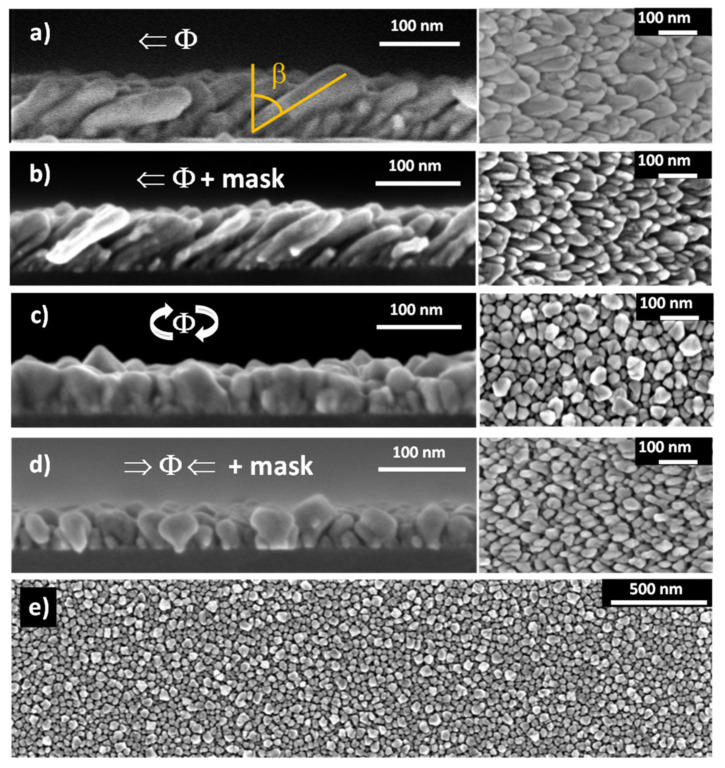
Cross-sectional (left column) and top-view (right column and bottom expanded image) SEM images of 100 min MS-GLAD Fe deposition onto Si substrate with *σ* = 85° of: (**a**) NR sample: no azimuthal rotation of the substrate; (**b**) NR-mask: no azimuthal rotation of the substrate and use of mask (one side deposition); (**c**) R sample azimuthal rotation of the substrate; and (**d**) R-mask: azimuthal rotation of the substrate and use of collimation mask (two sides deposition). (**e**) Large area top-view of (**c**) case.

Figure 2 depicts SEM images (top-view and cross-sectional) of the four samples obtained by MS-GLAD at different growth conditions. Each sample shows a distinct morphology, and the geometrical parameters obtained from the images analysis are summarized in Table 1. The cross-sectional images (left column) reveal that the nanostructures lateral shape corresponds to inclined or vertical nanopillars that start with a narrow diameter at the substrate and are enlarged after the initial nucleation. The film thickness of the NPs samples varies between 55 and 76 nm. Although the deposition time was kept constant for all samples (100 min), the 21 nm thickness variation among them is mainly determined by the presence or not of the mask collimating the atomic flux. Since the mask selects a portion of the sputtered atoms that leave the target at a given angle, the incorporation of thermalized and partially thermalized species is minimized. Consequently, fewer atoms reach the substrate [37,38].

The two MS-GLAD samples deposited without substrate rotation, NR (Figure 2a) and NR-mask one side deposition (Figure 2b), consist of tilted NPs. Even if the atomic flux arrives at the Si substrate with an incident angle of σ=85°, the NP tilt angle, *β*, measured relative to the substrate normal, is β=58° in both cases. It is well known that the nanostructure tilt angle obtained with MS-GLAD deposition does not correspond to the incidence angle of the atomic flux [39,40,41]. *β* depends on several factors such as deposited material (Fe, Ti, Ag, Au…) [40], substrate type [42] and its surface roughness [30], crystallinity of the deposited material [11], and growth parameters (rate [11], temperature [43,44], pressure [45], and plasma condition [34]). Tait et al. [41] found a phenomenological relation between the column tilt angle and the deposition angle as $2sin\left(\sigma -\beta \right)=1-\cos(\sigma )$ for which they assumed ballistic deposition and shadow effects. This expression yields to β=58° for σ=85°. The matching between the theoretical value and the experimental one demonstrates that the ballistic growth regime dominates in our working conditions.

The average diameter of the NPs in the NR sample (31 nm) is almost twice the average diameter of those in NR-mask sample (16 nm). The deposition through the mask induces the selective deposition of highly directed species, i.e., improves the collimation, which leads to narrower and better-defined tilted nanopillars with reduced coalescence in the direction perpendicular to the atomic flux ($\Phi_{perp}$).

In addition, in NR sample the higher coalescence in $\Phi_{perp}$ direction relative to the parallel one ($\Phi_{paral}$) (see Figure 2a-planar view) suggests an anisotropic density of NPs. In order to analyse this morphological anisotropy, the distance separating the tops of neighboring nanopillar pairs, both in the parallel and perpendicular directions, has been measured. The obtained average distances are 51.1 and 26.5 nm respectively. Therefore, the separation between NPs in the $\Phi_{perp}$ direction is approximately half that in the $\Phi_{paral}$ one and matches numerically the diameter of the thinnest NPs (the few isolated ones not exhibiting coalescence). Therefore these results point to an anisotropic NPs density, increased in the $\Phi_{perp}$ direction.

Vertical nanopillars are obtained if the deposition is carried out while the substrate rotates, either continuously without a mask, as in sample R (Figure 2c), either rotating 180° every 120 s when the mask is used, as in sample R-mask (Figure 2d). In both cases, the NPs do not show a cylindrical shape, but they broaden with increasing height. When the substrate rotates, the self-shadowing mechanisms of the nanocolumns is less effective; thus, the pillars become not only vertical but also wider, resembling an inverted pyramid. This phenomenon is also present in pillars fabricated by e-beam evaporation [14] but is obviously more significant with sputtering [46]. The main morphological difference between these two samples can be observed from the top-view images: all directions in the plane are equivalent in sample R, whilst sample R-mask has pillars with more elongated shape in the flux direction, as can be expected for two-sided alternating deposition obtained by rotating 180° the substrate.

The sample porosity has been calculated with respect to the saturation magnetization measured for the thin film ($M_{S}=1190\;\mathrm{emu}/{\mathrm{cm}}^{3}$). Size and surface effects affecting thin films at the nanoscale, in addition to the Fe oxidation, account for the reduction of $M_{s}$ relative to its bulk value $\left(M_{S,Fe}=1714\;\mathrm{emu}/{\mathrm{cm}}^{3}\right)$ [47]. As the magnetometer gives the total magnetic moment, by obtaini ng the sample lateral dimensions from high-quality photographs and their thickness from cross-section SEM images, the saturation magnetization $M_{s}$ can be deduced. If we assume that the measured magnetic moment does not vary between the normal incidence sample and those deposited in MS-GLAD configuration [31], the effective volume and hence the porosity of the MS-GLAD samples can be estimated by comparing their respective $M_{s}$ values. The porosity percentages shown in Table 1 vary between 28% and 69%, which are reasonable values compared to previous results obtained in gold nanopillars [35]. These results evidence how the porosity can be adjusted with the growth conditions. The highest porosity is achieved when the substrate is not rotating azimuthally during the deposition (NR and NR-mask samples), i.e., for tilted NPs. In particular, the NR-mask sample presents the highest porosity value, due to the improved atom collimation that induces better NP definition and larger separation between them. On the opposite extreme is the R sample, with the lowest porosity, in agreement with the rotation and the absence of mask, which produce the poorest collimation of all the analyzed cases.

Finally, Figure 2e illustrates an example of how homogeneous are the nanostructures that can be produced by MS-GLAD, regardless of the particular growth conditions.

**Figure 3:**
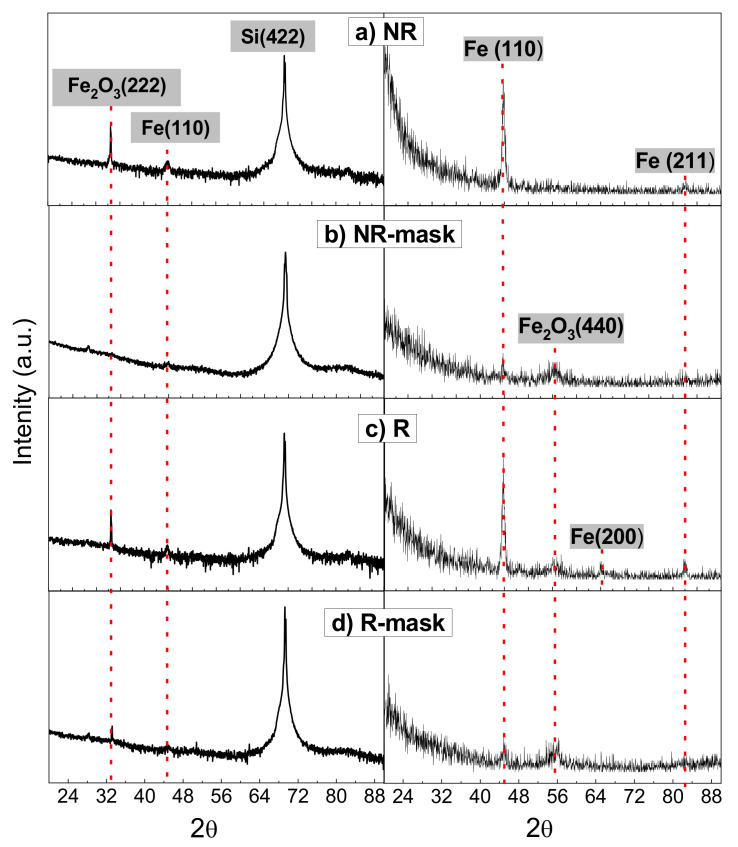
XRD diagrams in Bragg-Brentano configuration (left column) and Grazing Incidence configuration at 0.5° with respect to the substrate plane (right column) for: (**a**) NR sample: no azimuthal rotation of the substrate; (**b**) NR-mask: no azimuthal rotation of the substrate and use of mask (one side deposition); (**c**) R sample azimuthal rotation of the substrate; and (**d**) R-mask: azimuthal rotation of the substrate and use of collimation mask (two sides deposition).

The structural properties and composition of the samples investigated by XRD measurements are shown in Figure 3. In the conventional Bragg-Brentano configuration (left column of Figure 3), weak signals are measured due to the small film thickness. Apart from the silicon peak at 2*θ* = 69.4° assigned to Si(422), there are two peaks at 2*θ* = 33.1° and 44.9° that correspond to Fe_2_O_3_(222) and α-Fe(110), respectively, with iron oxide being dominant. As expected, a portion of un-capped Fe NPs develops into Fe_2_O_3_ in air atmosphere.

In contrast, more prominent peaks associated with Fe are detected in Grazing Incidence configuration performed at 0.5° (see right column of Figure 3). In this configuration, the peak at 2θ=44.9° (α-Fe(110)) is predominant in the XRD diffraction pattern. In addition, other peaks related to the α-Fe structure are visible (see Figure 3c for R sample): 2θ=65.1° assigned to α-Fe(200) and 2θ=82.2° to α-Fe(211) (ICDD 01-087-0721). Finally, in this geometry, the presence of Fe_2_O_3_ is evidenced by the peak at 2θ=55.4° that corresponds to Fe_2_O_3_(440). On the other hand, it is not easy to determine the phase type of Fe_2_O_3_ from a single peak at 2*θ* = 33.1° measured in Bragg-Brentano configuration and a single peak at 2*θ* = 55.4° measured in Grazing Incidence configuration. While the first reflection is the most characteristic of hematite (α-phase), being absent in maghemite (γ-phase), the presence of the second reflection at 55.4° seems to indicate that this oxide may be in its cubic phase *β*(ICDD 04-003-1027).

Grazing incidence minimizes the substrate contribution in the patterns allowing for better detection of the film compound crystallinity. Even though the MS-GLAD is performed at room temperature, for which no long-range crystallinity is expected, the four samples are crystalline. A crystalline coherent size of d~15.5 nm from 2θ=44.9° (α-Fe(110)) reflection in both NR and R samples has been estimated with the Scherrer equation. The polycrystalline character of these samples can also be inferred from the comparison of the crystalline coherent size with the NP average length, L~125 nm and L~60 nm for NR and R, respectively (see Table 1).

### 3.2. Hysteresis Loops

**Figure 4:**
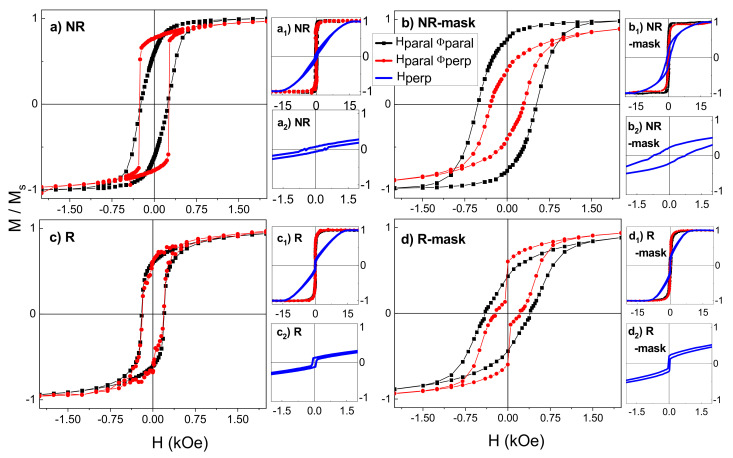
Normalized magnetization curves with the magnetic field applied parallel to the substrate surface (in-plane) and along the atomic flux projection direction ($H_{paral}\Phi_{paral}$; black curves) and perpendicular to it ( $H_{paral}\Phi_{perp}$; red curves) for samples: (**a**) NR; (**b**) NR-mask; (**c**) R, and (**d**) R-mask. Normalized saturated magnetization curves measured in-plane sample surface and perpendicular to it for: (**a1**) NR; (**b1**) NR-mask; (**c1**) R; and (**d1**) R-mask. Zoom of magnetization curves with perpendicular field for: (**a2**) NR; (**b2**) NR-mask; (**c2**) R; and (**d2**) R-mask.

In order to study the nanopillars magnetic behavior, room temperature magnetization measurements were carried out with magnetic applied field both in the substrate plane ($H_{paral}$) and perpendicular to it ($H_{perp})$. In the case of in-plane configuration, $H_{paral}$ was applied either parallel and perpendicular to the projection of the atomic flux in the film plane: $H_{paral}\Phi_{paral}$ and $H_{paral}\Phi_{perp}$, respectively. Figure 4(a1–d1) show the saturated hysteresis loops of the four samples measured with the magnetic field applied along the three directions: $H_{paral}\Phi_{paral}$, $H_{paral}\Phi_{perp}$, and $H_{perp}$, whereas Figure 4a–d and Figure 4(a2–d2) present a closer look at the center of the hysteresis loops measured with $H_{paral}$ (both directions) and $H_{perp}$, respectively. In Figure 4 the magnetization has been normalized to its value at 2 T since this field ensures a saturation state for all sample configurations.

The coercivity ($H_{C}$), saturation magnetization ($M_{s}$), anisotropy field $\left(H_{K}\right)$ and remanence-to-saturation magnetization ratio ($M_{r}/M_{s}$) derived from the hysteresis curves are displayed in Table 2. Special attention should be taken with the $M_{s}$ value. The volume considered for the normalization of the total momentum of each sample (measured in emu) was that of the hypothetical compact thin film associated with the geometrical dimensions of the substrate and the thickness of the film shown in the first column of Table 1 (i.e., without considering the porosity). Therefore, as this volume is overestimated with respect to that of the real porous NPs films, the $M_{s}$ values shown in Table 2 are underestimated. Only in the case of the thin film, the $M_{s}$ reported relates to its real value. As discussed in Section 3.1, from the comparison between the $M_{s}$ of the thin film and the underestimated $M_{s}$ of the NPs samples in Table 2, the porosity of each sample shown in Table 1 was calculated.

Depending on both the applied field direction and the NPs deposition conditions, the coercive field ranges from 35 to 790 Oe; the saturation field along the hard axis ($H_{K}$) varies between 1 T and 1.8 T; and the squareness, $M_{r}/M_{s}$, ranges between 0.01 and 0.8. Therefore, a very wide variability of the magnetic response can be achieved by modulating the NP morphology.

The critical radius for the multi-domainto single-domain transition for Fe nanoparticles varies between 3 and 25 nm depending on the magnetocrystalline anisotropy strength. Considering the NPs dimensions with average diameters ranging between 14 and 41 nm and average lengths between 55 and 125 nm, the nanopillars are expected to be in a magnetic multidomain state [48].

The magnetic parameters of a Fe thin film grown by sputtering with conventional geometry, i.e., at normal incidence, are included as a reference. It presents a soft and isotropic in-plane magnetic behavior with $H_{C}=15$ Oe and $M_{r}/M_{s}\sim 1$. However, when the magnetic field is applied perpendicular to the substrate, $H_{C}$ increases to 375 Oe, $M_{r}/M_{s}$ decreases to 0.18, and $H_{K}$ is the highest listed in Table 2 (1.8 T).

Focusing our attention on the NP magnetic behavior, the first thing to note is the magnetic hardening achieved with the nanopillars morphology as compared to the film. The highest in-plane coercivity measured in NPs (522 Oe for NR-mask sample) is ~35 times greater than that measured for the thin film. Even the smallest one (189 Oe) is ~13 times the thin film coercivity.

On the other hand, regardless of the fact that the NPs geometrical axis is out of the plane (either tilted for the NR and NR-mask samples or vertical for the R and R-mask ones), the magnetization easy direction has a predominant component in the substrate plane as can be inferred from $M_{r}/M_{s}$ values in Table 2 and from the fields required to saturate the magnetization in the in-plane and perpendicular directions. Typically, one would expect the magnetization to be aligned more easily along the pillar geometric axes due to the shape anisotropy of individual NPs. It is particularly remarkable that this is not the case for samples with vertical nanopillars (R and R-mask). In these two cases, a field in the range of 3000–4000 Oe was required to saturate the magnetization in the in-plane direction while a field as high as 10,000–15,000 Oe is needed to saturate the samples in the out-of-plane direction. The reduced thickness of the films containing vertical or tilted NPs and their close-packed configuration contributes to a strong demagnetizing effect perpendicular to the substrate due to the dipolar-like interactions among neighbouring nanopillars, as it happens in vertical electrodeposited nanowires [49]. Actually, the shape of the loop in the vertical direction for the R and R-mask samples is similar to that obtained for Liu and co-workers for Fe columns with micrometer length (see Figure 1d in [14]). In addition, a continuous layer is always formed near the substrate in the early stages of growth. Indeed, a minimum amount of deposited material is required till the highly directional deposition particle flow develops the surface mounds that act as efficient seeds for the formation of nanopillars. As a result, the whole system is composed of a continuous irregular layer (only a few nm thick but without homogeneous height) covered by nanopillars. This fact has been demonstrated by cross-sectional transmission electron microscopy in other works [30].

Nevertheless, the vertical morphology of the nanopillars, obtained when substrate rotation is used, plays a significant role on the loop shape and coercivity with $H_{perp}$ (see Table 2 and Figure 4(a2–d2): in R and R-mask the loops exhibit a square shape around zero field and $H_{C}$ in the perpendicular direction is one order of magnitude smaller than for tilted NPs and thin film. In addition, the anisotropy field for vertical NPs is smaller than for the tilted NPs, especially when the mask is used (sample R-mask).

On the other hand, although NR and NR-mask have been fabricated under the same growth conditions, the process of growing one of them through a mask has a marked influence on the magnetic response (see Figure 4a,b for comparison).

Beginning with NR sample, Figure 4a shows that in $H_{paral}\Phi_{perp}$, the magnetic reversal occurs suddenly and with a higher $M_{r}/M_{s}$ as compared to the $H_{paral}\Phi_{paral}$ case (Table 2), even though the coercivity remains unchanged in both field directions. The simultaneous reversal of the magnetization in the in-plane direction perpendicular to the atomic flux suggests either a high degree of magnetic correlation between the NPs or an in-plane easy axis perpendicular to the atomic flux. In contrast, in the in-plane direction parallel to the atomic flux, the magnetization reversal takes place gradually, i.e., is a NP-size dependent process. It follows that in this sample, the shape anisotropy of the individual NPs is not the determining factor of the magnetic response.

The mentioned in-plane anisotropy could be an extra growth-induced uniaxial anisotropy term. It has long been reported [9,10,50] that oblique deposition configuration would induce an in-plane uniaxial magnetic anisotropy typically perpendicular to the deposition direction. The shadow effect and the limited surface diffusion promote the formation of columns that tend to coalesce perpendicular to the incoming atomic flux, thus generating an in-plane texture in this direction ($H_{paral}\Phi_{perp}$), which corresponds to the film easy axis. This anisotropic geometry induced by the shadowing effect was first observed by Konig and Helwig [51] in GLAD incidence films of Al, Pt, and tungsten oxide by electron microscopy. The same effect was reported with both magnetic [12,19,20,21,26,32] and non-magnetic [6,52] materials over the years. In fact, for the NR and R-mask samples, a closer look at the zenithal SEM images (i.e., Figure 2a,d) top view) suggests that NPs are not distributed homogeneously on the surface but with increased porosity and relatively less dense film along the direction parallel to the atomic flux.

In the case of the NR-mask sample, the highest values of coercivity (522 Oe, 295 Oe, and 791 Oe measured with $H_{paral}\Phi_{paral}$, $H_{paral}\Phi_{perp}$, and $H_{perp}$, respectively) and a smaller magnetic correlation in the magnetization reversal (i.e., less abrupt changes in the loop) in both in-plane field directions are observed (Figure 4b). The mask promotes the formation of NPs magnetically isolated from each other (except for the initial layer that connects them at the base), i.e., without coalescence in the direction perpendicular to the flux. The NPs exhibit enhanced shape definition and smaller diameter, resulting in a higher length/diameter ratio. This fact induces an increase in the out of plane remanent magnetization, as shown in Table 2 when comparing $M_{r}/M_{s}$ in the $H_{perp}$ direction for the NR and the NR-mask samples. In addition, regarding the in-plane behavior of the films, unlike in NR, $M_{r}/M_{s}$ for NR-mask is higher when the in-plane field is applied parallel to the atomic flux than when it is perpendicular to it. All these features indicate that its magnetic response is determined mainly by the interplay between the shape anisotropy of individual NPs and the extra growth-induced uniaxial anisotropy rather than by strong magnetic interactions. This will be confirmed by the micromagnetic simulations detailed in Section 4.

Regarding the samples with vertical nanostructures, the R sample shows an almost isotropic in-plane magnetic behavior (Figure 4c) as expected for vertical nearly cylindrical NPs that were grown without any preferential in-plane direction due to the substrate rotation.

Finally, the R-mask sample (see Figure 4d) presents an anisotropic in-plane behavior. The easy axis of R-mask is along $H_{paral}\Phi_{perp}$ as in the tilted NR case. The particular deposition method for R-mask, with the incoming atoms arriving only from two opposite directions in an alternate way, generates more contact between the elongated cones across $\Phi_{perp}$ than across $\Phi_{paral}$. In addition, a bimodal distribution of coercivity is observed in $H_{paral}\Phi_{perp}$. The morphology of R-mask, truncated cones with a bottom smaller diameter than the top, could result in two different coercivities. Nevertheless, if the singular morphology of a single cone was the determining factor of the bimodal coercivity, the same feature would be present in the $H_{paral}\Phi_{paral}$ direction, which is not the case. As will be discussed later, such bimodal coercivity is due to the connection of the pillars along the short-axis direction $\left(H_{paral}\Phi_{perp}\right)$, which acts as an effective magnetic anisotropy.

### 3.3. FORC Diagrams

More information about the nanopillar magnetic behavior can be extracted from the FORC distributions measured in-plane ($H_{paral}$) both in the parallel and perpendicular directions with respect to the projection of the atomic flux. We focused on the consequences of the flux collimation and the nanopillar coalescence on the magnetization behavior (NR and NR-mask samples). Note that all FORC measurements discussed here exhibit an important reversible behavior, denoting some coherent magnetization reversal. However, this contribution does not appear in the FORC diagrams, which only represent the signature of the irreversible mechanisms.

**Figure 5:**
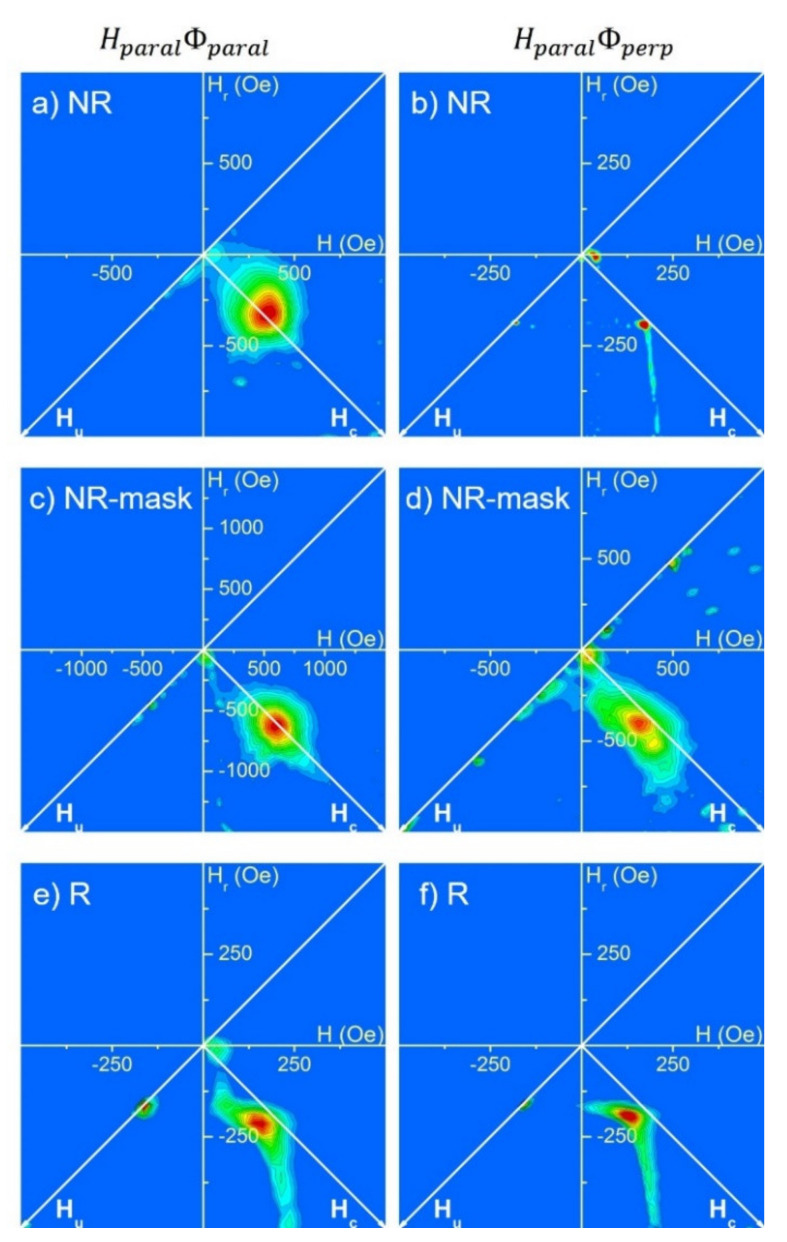
FORC distributions measured with the in-plane magnetic field parallel to the atomic flux ($H_{paral}\Phi_{paral}$, left column) and perpendicular to it ( $H_{paral}\Phi_{perp}$, right column) for: (**a**) and (**b**) NR sample; (**c**) and (**d**) NR-mask sample; and (**e**) and (**f**) R sample.

The system porosity affects the magnetic behavior in both in-plane directions for tilted nanopillars. For both samples (NR and NR-mask), which also present the highest porosity, the parallel FORC results ($H_{paral}\Phi_{paral}$) are compatible with a behavior of independent entities mainly governed by the shape anisotropy (Figure 5a,c). However, by comparing the distributions, we observe that a higher porosity and better nanopillar definition yields an elongation of the FORC distribution along the coercivity axis ($H_{c}$) (NR-mask, Figure 5c). This elongation arises from a coercivity distribution among the nanopillars and can be explained by their geometric differences. Without collimating the atomic flux (NR, Figure 5a), the circular FORC distribution indicates a more uniform coercivity among the nanopillars, even if they are also with some geometrical differences (see Figure 2a). Remembering that the parallel direction is along the tilted nanopillar axis, these results suggest that the small ellipticity naturally occurring from the shadow effect in NR (as will be considered in the micromagnetic simulations, Section 4) sufficiently influences the magnetization reversal. Both the narrower FORC coercivity distribution and lower average coercivity (also observed from hysteresis loops) may arise from a reversal not governed by the shape anisotropy, as in the NR-mask case.

As expected, the nanopillar coalescence in the direction perpendicular to the flux strongly affects the magnetic behavior of these tilted NPs when the magnetic field is applied along that direction $\left(H_{paral}\Phi_{perp}\right)$, see Figure 5b,d. For independent NPs (NR-mask, Figure 5d), applying a magnetic field perpendicular to the nanopillar axis yields a considerable reversible behavior noticeable at the FORCs beginning (not shown). Even if the magnetization reversal is not completely coherent, as could be expected for ideal nanopillars, the lower remanence and coercivity compared to the parallel direction (see Figure 4b) support this hypothesis. The large FORC distribution elongation along the $H_{c}$ axis is compatible with nanopillars geometrically different and disconnected from their neighbors. On the other hand, when the nanopillars present coalescence, the FORC distribution drastically changes (NR, Figure 5b). Its corner shape can be attributed to a system with a large coercivity distribution, but where a positive retroaction occurs during the magnetization reversal. This phenomenon, where the switching initiation favors the reversal of the rest of the magnetization, is attributed to the direct contact among the neighboring nanopillars and induces an additional uniaxial perpendicular anisotropy, as previously discussed in Secttion 3.2 based on the hysteresis loop.

Finally, for vertical nanopillars in R sample, the in-plane magnetic behavior is isotropic. The low flux collimation during fabrication in this case induces a porosity lower than for NR and NR-mask samples. Observing the R sample FORC diagrams, no noticeable difference appears among both measurement directions $H_{paral}\Phi_{paral}$ and $H_{paral}\Phi_{perp}$ (Figure 5e,f, respectively). Interestingly, both distributions exhibit a corner shape, although less pronounced than for tilted and coalesced nanopillars (NR sample in $H_{paral}\Phi_{perp}$ configuration). This suggests that, for the sample fabricated with azimuthal rotation of the substrate but without mask, the low flux collimation and limited surface diffusion induce some interconnections between the nanopillars. Since these coalescences, which do not exhibit a preferred direction, are only partial, they yield a weaker positive retroaction during the magnetization reversal.

In conclusion, the FORC results show that the improved flux collimation obtained when a mask is used during deposition induces a magnetic behavior of independent nanopillars, governed by their individual shape anisotropy and thus with a large distribution of coercive fields. Removing this collimation yields the coalescence of some nanopillars, resulting in an avalanche-like magnetization reversal. This interconnection can be anisotropic (perpendicular to the flux), such as in the NR sample, or isotropic, as in the R sample case.

## 4. Micromagnetic Simulations and Discussion

### 4.1. Micromagnetic Simulations

**Figure 6:**
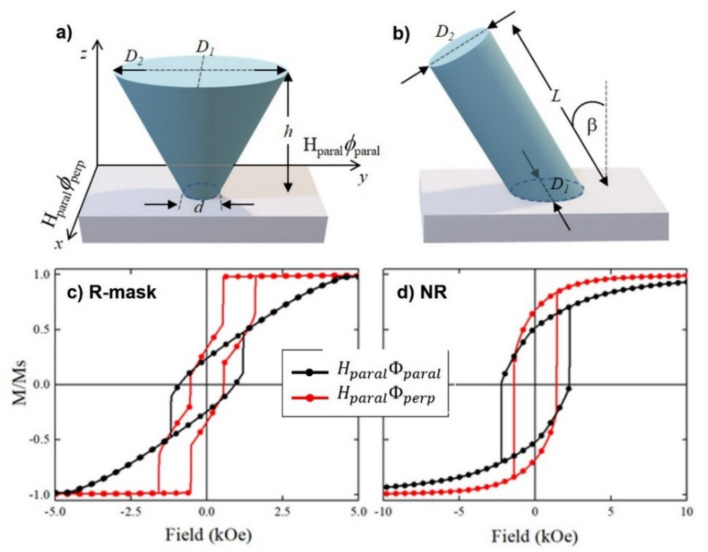
Diagram and geometric parameters of the nanostructures used to model the (**a**) R-mask sample and the (**b**) NR sample in the micromagnetic simulations. Simulated hysteresis loops for R-mask (**c**) and NR (**d**) samples when a magnetic field is applied parallel ($H_{paral}\Phi_{paral}$, black dots) and perpendicular ( $H_{paral}\Phi_{perp}$, red dots) to the direction of the atomic flux projection during deposition (*y*-axis of the diagram) and with an uniaxial anisotropy perpendicular to the flux direction (*x*-axis) with a magnitude of $K_{u}=600\;\mathrm{kJ}/m^{3}$.

To identify the critical parameters of the nanopillar systems determining their magnetic behavior, we performed several micromagnetic simulations in two representative samples, R-mask (see Figure 2d) and NR (see Figure 2a), while adding some characteristics observed experimentally. We have modeled R-mask and NR samples as truncated elliptical cones and inclined elliptical columns, as schematically represented in Figure 6a,b, respectively. For both samples, the dimensions of a representative nanostructure have been selected. For R-mask, *D*_1_ = 30 nm, *D*_2_ = 52.5 nm, *d* = 17.5 nm, and *h* = 55 nm, while for NR *D*_1_ = 22 nm, *D*_2_ = 26 nm, *L* = 125 nm, and *β* = 58°. We have considered cell sizes of 2 × 2.5 × 2.0375 nm^3^ for R-mask and 1 × 1 × 2.5 nm^3^ for NR sample. Finally, we have applied an in-plane external magnetic field parallel ($H_{paral}\Phi_{paral}$) and perpendicular ($H_{paral}\Phi_{perp}$) to the y-axis of the system (Figure 6a) on a uniformly saturated sample in the $H_{paral}\Phi_{paral}$ and the $H_{paral}\Phi_{perp}$ direction, respectively, following the experimental measurement procedures.

**Figure A1:**
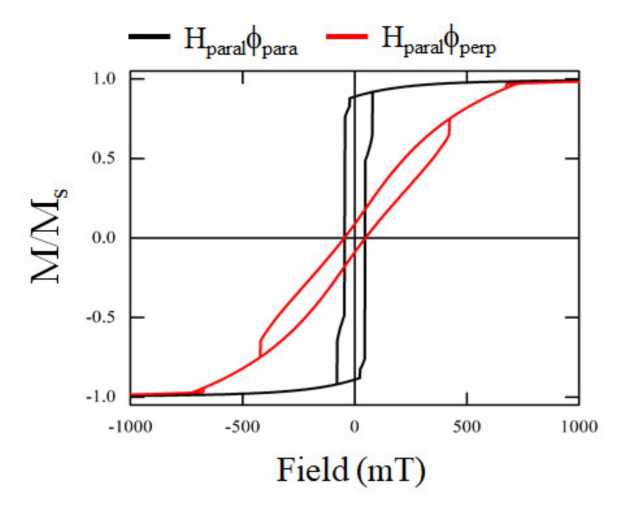
Hysteresis loops for an isolated conical nanostructure with *D*_1_ = 30 nm, *D*_2_ = 52.5 nm, and *h* = 55 nm in the absence of magnetocrystalline anisotropy when a magnetic field is applied parallel ($H_{paral}\Phi_{paral}$, black line) and perpendicular ( $H_{paral}\Phi_{perp}$, red line) to the *y*-axis of the system.

**Figure A2:**
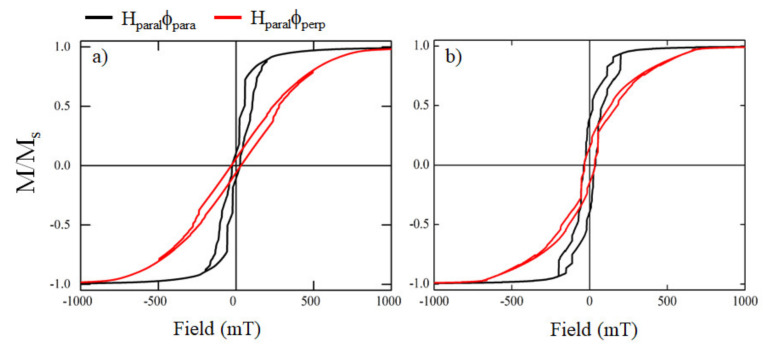
Hysteresis loops for an array of seven conical nanostructures with *D*_1_ = 30 nm, *D*_2_ = 52.5 nm, *h* = 55 nm, and center-to-center distance of 60 nm in the absence of magnetocrystalline anisotropy when a magnetic field is applied parallel ($H_{paral}\Phi_{paral}$, black line) and perpendicular ( $H_{paral}\Phi_{perp}$, red line) to the *y*-axis of the system. (**a**) corresponds to the array without base and (**b**) corresponds to the array supported on a 4.5 nm thick iron base.

In the first run, we simulated isolated nanostructures in the absence of magnetocrystalline anisotropy in order to consider only the effect of shape anisotropy, neglecting the magnetostatic interaction between NPs (see Figure A1 in Appendix A). As the results did not reproduce the experimental results, in a second run we simulated a hexagonal array of seven of these nanostructures to investigate the role of the magnetostatic interactions, without and with the iron base that supported the nanostructures (see Figure A2a,b in Appendix A, respectively). However, all these oversimplified systems do not reproduce the experimental results, indicating that nor the magnetostatic interactions nor the base connection between the nanopillars governed their reversal behavior. Therefore, in a last series, we introduced a uniaxial anisotropy in the $H_{paral}\Phi_{perp}$ direction that we adjusted until reproducing the experimental results.

**Figure A3:**
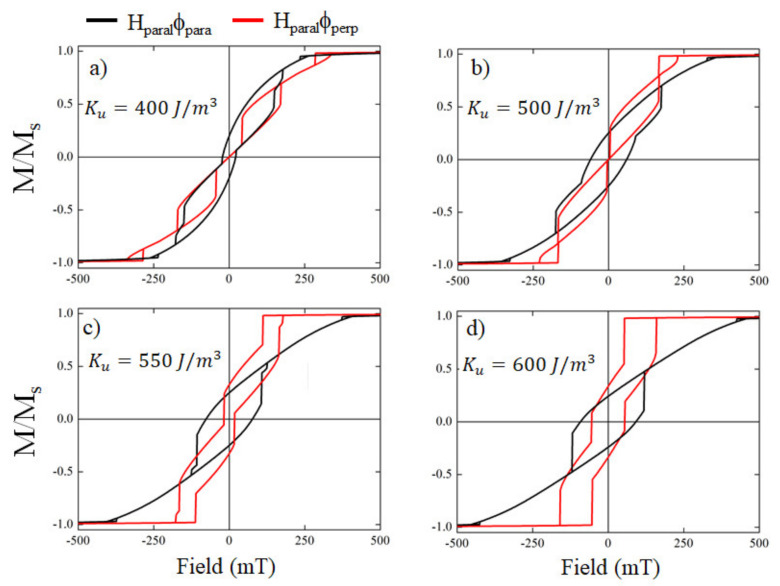
Hysteresis loop of a single sample R-mask when a uniaxial anisotropy of magnitude (**a**) *K*_u_ = 400 J/m^3^, (**b**) *K*_u_ = 500 J/m^3^, (**c**) *K*_u_ = 550 J/m^3^and (**d**) *K*_u_ = 600 J/m^3^, is induced in the $H_{paral}\Phi_{perp}$ direction.

We have numerically investigated the hysteresis curves of the R-mask and NR samples, systematically varying the uniaxial magnetic anisotropy constant along the $H_{paral}\Phi_{perp}$ direction from $K_{u}=200$ to $600\;\mathrm{kJ}/m^{3}$, comparing with the experimental results (see Figure A3 in Appendix A). The best agreement has been obtained when we use a uniaxial anisotropy parallel to the *x*-axis with a magnitude of $K_{u}=600\;\mathrm{kJ}/m^{3}$ (see Figure 6c,d).

**Figure 7:**
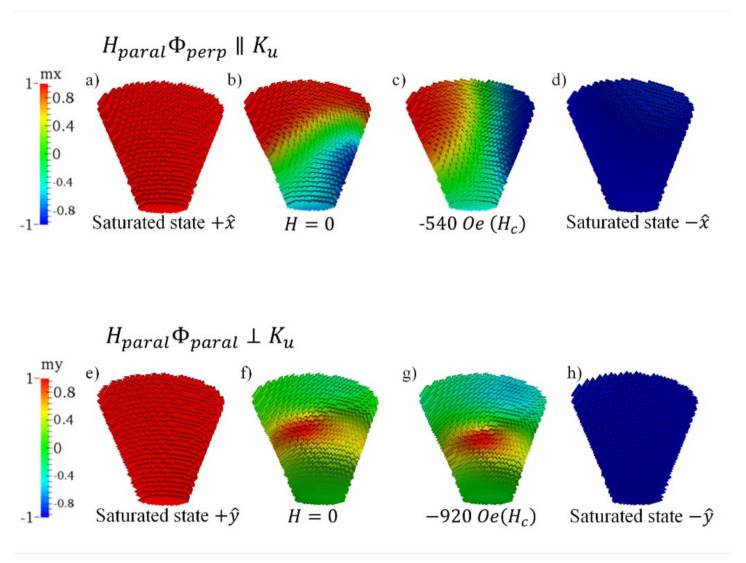
Snapshots of the stable magnetization state corresponding to the R-mask sample. $K_{u}$ is the uniaxial magnetic anisotropy constant in the $H_{paral}\Phi_{perp}$ direction. Top row is for magnetic field applied along the $H_{paral}\Phi_{perp}$ direction with different values: (**a**) *H* = 25,000 Oe (i.e., saturated state in the positive direction), (**b**) *H* = 0, (**c**) *H* = −540 Oe, (**d**) *H* = −25,000 Oe (i.e., saturated state in the negative direction). Bottom row is for magnetic field applied along the $H_{paral}\Phi_{paral}$ direction with different values: (**e**) *H* = 25,000 Oe (i.e., saturated state in the positive direction), (**f**) *H* = 0, (**g**) *H* = −920 Oe, (**h**) *H* = −25,000 Oe (i.e., saturated state in the negative direction).

**Figure 8:**
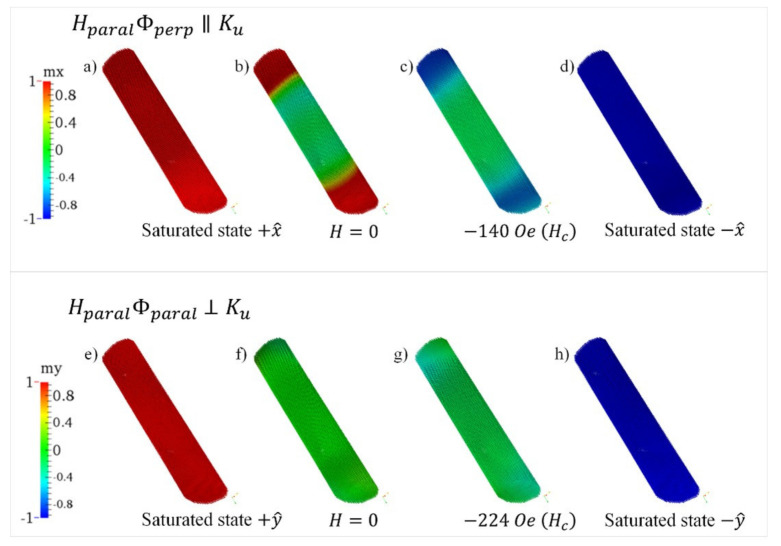
Snapshots of the stable magnetization state corresponding to the NR sample. Top row is for magnetic field applied along the $H_{paral}\Phi_{perp}$ direction with different values: (**a**) H = 25,000 Oe (i.e., saturated state in the positive direction), (**b**) *H* = 0, (**c**) *H* = −140 Oe, (**d**) *H* = −25,000 Oe (i.e., saturated state in the negative direction). Bottom row is for magnetic field applied along the $H_{paral}\Phi_{paral}$ direction with different values: (**e**) *H* = 25,000 Oe (i.e., saturated state in the positive direction), (**f**) *H* = 0, (**g**) *H* = −224 Oe, (**h**) *H* = −25,000 Oe (i.e., saturated state in the negative direction).

To better understand the magnetization reversal mechanism of these systems, in Figure 7 and Figure 8 we show snapshots of the magnetization for samples R-mask and NR, respectively. R-mask sample is simulated as a truncated elliptical cone (see Figure 6a). When the magnetic field is applied parallel to the flux direction or *y*-axis ($H_{paral}\Phi_{paral}$), see Figure 7 bottom row, the system reverses its magnetization by coherently rotating the spins at the ends of the cone. The spins on the bottom cover rotate towards −*x* to reach −*y*, while the spins on the top cover rotate in the opposite direction, turning towards +*x* to reach −*y*. This process is reflected in a continuous change in the magnetization of the sample in the hysteresis curve. Only the spins located in the center of the cone remain pinned and require a greater magnetic field to produce their reversal, a fact that is reflected in the abrupt jump of the hysteresis curve.

On the other hand, when the field is applied in the direction perpendicular to the flux or *x*-axis ($H_{paral}\Phi_{perp}$), see Figure 7 top row, the system exhibits a completely different hysteresis curve, with well defined abrupt jumps. In this case, the process of magnetization reversal begins with the lower cover, with a smaller diameter, giving rise to two well-defined domains, the lower one where the spins point towards −*x* and the upper one with the spins pinned towards +*x*. Both domains are separated by a domain wall, which begins to rotate until it is completely vertical along the axis of the cone, stabilizing an internal vortex. Obviously, the vortex is very stable, and it requires a much larger field to bring the spins towards −*x*, which explains the last Barkhausen jump that describes the hysteresis curve.

The NR sample is simulated as an inclined elliptical column (see Figure 6b). In this case, when the magnetic field is applied parallel to +*x* ($H_{paral}\Phi_{perp}$), see Figure 8 top row, the system begins by reversing the spins in the central area of the pillar by coherent rotation until they are pointed parallel to the column axis, which is reflected in a continuous decrease in the magnetization of the hysteresis curve. Then the spins pinned in the ends abruptly reverse, producing a sharp magnetization jump in the hysteresis curve. Finally, the spins in the central zone continue rotating until they point towards −*x*.

On the other case, when the magnetic field is applied parallel to the *y*-axis ($H_{paral}\Phi_{paral}$), see Figure 8 bottom row, the system reverses in a way very similar to that described above, that is through coherent rotation. However, the area that maintains spins pinned in the covers is much lower in this configuration, yielding a smaller jump.

### 4.2. Discussion

The difference between in-plane hysteresis loops along $\Phi_{perp}$ and $\Phi_{paral}$ indicated that an in-plane uniaxial anisotropy exists in some samples. Due to the polycrystalline character of the samples and thus the absence of a significant magnetocrystalline anisotropy, the magnetization direction is largely influenced by morphological effects. In fact, compared to the thin film, pillar structures obtained in MS-GLAD geometry have a crucial role in controlling the magnetic properties of the arrays due not only to the shape of the individual pillars but also to possible anisotropic features. This latter effect is precisely what occurs in the NR and R-mask samples, in which the distance between neighboring nanopillars is shorter along $\Phi_{perp}$ than along $\Phi_{paral}$. In other words: the arrays are less compact along the flux direction ($\Phi_{paral}$) than along the perpendicular one ($\Phi_{perp}$). Actually, coalescence along the $\Phi_{perp}$ direction can even be observed in different locations in the top-view SEM images of those samples (see the images on the right of Figure 2a,d). This phenomenon was called “bundle formation” by Hara and co-workers [12]. It represents the origin of the additional in-plane uniaxial magnetic anisotropy perpendicular to the atomic flux. This anisotropy was crucial in the micromagnetic simulations in order to reproduce the experimental in-plane hysteresis loops (see Figure 6c,d).

This effective uniaxial magnetic anisotropy has been reported in other works. With samples fabricated by thermal evaporation, Bubbendorf et al. found it in obliquely deposited Fe films [22] and Morrow et al. also described it in Co/Cu multilayered slanted nanopillars [24]. In the case of sputtering, Schlage et al. observed similar behavior in 5 nm thick polycrystalline iron films prepared with 80° tilt angle [25] and Wang et al. reported analogous anisotropy in permalloy films obliquely deposited at 50° [18].

Having this in mind, now we can resume the discussion at the end of Section 3.2, i.e., the bimodal coercivity observed for the in-plane hysteresis loop of sample R-mask along the perpendicular-to-flux direction. Such loop is the result of the existence of two easy axes in this sample: one in the plane due to the effective anisotropy discussed above along the perpendicular-to-flux direction, and another perpendicular to the substrate due to the inverted cone shape of each independent nanopillar. So, the first step of the loop corresponds to the magnetization switching from the positive $\Phi_{perp}$ direction to the vertical one, whereas the second step is the switching from the vertical direction to the negative $\Phi_{perp}$ direction. It is worth mentioning that this kind of hysteresis loop with two steps when the field is applied along the $\Phi_{perp}$ direction has been also reported by Morrow et al. [24] and by Wang et al. [18].

## 5. Conclusions

To conclude, we have presented a method to tune morphological and magnetic properties of Fe- based nanostructures (Fe and Fe_2_O_3_) in large area by changing the growth conditions in glancing deposition geometry. Size, shape, tilt angle and inter-pillar separation can be tailored by choosing the appropriate conditions. For tilted NPs fabricated by MS-GLAD, an enhanced coalescence is observed in the direction perpendicular to the atomic flux (the short NPs axis) which has a significant impact on their magnetic behavior: Their magnetic easy axis is along their short morphological axis. Micromagnetic simulations reproduced the experimental shape of the hysteresis loops and concluded that the small inter-pillar separation in the direction perpendicular to the incoming atomic flux determines a growth-induced uniaxial magnetic anisotropy with magnetostatic origin and with an anisotropic constant of around $600\;J\;m^{-3}$.

The growth performed through a collimating mask mounted onto the substrate without azimuthal rotation produces better defined NPs with distinct magnetic properties. Both a reorientation of the in-plane magnetic easy axis and a hardening in the reversal process are measured in these well-separated NPs. It should be noted that the implementation of an atomic flow collimating mask prevents the developing of the extra in-plane uniaxial anisotropy term perpendicular to the incoming atomic.

On the other hand, vertical NPs in-plane magnetically isotropic are obtained if the glancing deposition is carried out while the substrate is rotated azimuthally and without the collimating mask.

Finally, if the substrate is alternatively rotated azimuthally so that the atomic flow can penetrate through the two opposite apertures of the mask, nanostructures in the form of inverted cones are produced. This singular morphology exhibits a bimodal distribution of coercivity when the field is applied in-plane and perpendicular to the atomic flux direction which is also a consequence of the growth-induced uniaxial anisotropy.

Therefore, the presents results demonstrate the potential of the MS-GLAD method to tailor the structural and magnetic material properties for technological applications.

## Figures and Tables

**Table 1:** Growth conditions and morphological parameters of Fe nanopillars (NPs) and thin film reference samples: Average values of film thicknesses (t), porosity (P), NP tilt angle (β), lengths (L) and diameters (D). t, β, L and D are measured from the cross-sectional SEM images (Figure 2a–d).

	Particular Growth Conditions	t (nm)	P (%)	β (°)	L (nm)	D (nm)	
NR	No azimuthal rotation of the substrate	76	50	58	125	31	
NR-mask	No azimuthal rotation of the substrate and use of mask (atoms coming from one side)	59	69	58	87	16	
R	Azimuthal rotation of the substrate	60	28	0	60	33	
R-mask	Azimuthal rotation of the substrate and use of mask (atoms coming from the two sides, alternatively)	55	42	0	55	*D*_top_ = 41	*D*_base_ = 14
Thin film	Not GLAD	25	0	-	-	-	-

**Table 2:** Coercivity ($H_{C}$ ), underestimated saturation magnetization ($M_{s}$), anisotropy field $\left(H_{K}\right)$ and remanence-to-saturation magnetization ratio $\left(M_{r}/M_{s}\right)$ for the NP samples and the reference thin film. $H_{C}$ and $M_{r}/M_{s}$ are evaluated for the three directions of the magnetic applied field: 1—Parallel both to the substrate and to the atomic flux projection ($H_{paral}\Phi_{paral}$), 2—Parallel to the substrate and perpendicular to the atomic flux projection ($H_{paral}\Phi_{per}$), and 3—Perpendicular to the substrate ($H_{perp}$).

	$H_{C}\;(\mathrm{Oe})$			$M_{s}\;(\mathrm{emu}/{\mathrm{cm}}^{3})$	$H_{K}\;(\mathrm{Oe})$	$M_{r}/M_{s}$		
	$H_{\mathrm{paral}}\Phi_{perp}$	$H_{\mathrm{paral}}\Phi_{perp}$	$H_{perp}$			$H_{\mathrm{paral}}\Phi_{\mathrm{paral}}$	$H_{\mathrm{paral}}\Phi_{perp}$	$H_{perp}$
NR	238	263	415	592	17,900	0.60	0.77	0.006
NR-mask	522	295	791	363	16,000	0.76	0.40	0.22
R	198	189	85	856	15,000	0.60	0.60	0.13
R-mask	400	215	35	683	10,100	0.43	0.60	0.21
Thin Film	15	15	375	1190	18,000	0.95	0.95	0.18

## Data Availability

Data presented in this article are available at request from the corresponding author.

## Appendix A. Micromagnetic Simulatioms

To reproduce the experimental results of the R-mask sample, we have systematically varied both the system and its geometric parameters, taking into account the following considerations:

### Appendix A.1. Isolated Conical Nanostructure without Magnetocrystalline Anisotropy

In a first series of simulations, we considered an isolated conical nanostructure (neglecting interaction with other nanostructures) with *D*_1_ = 30 nm, *D*_2_ = 52.5 nm, and *h* = 55 nm in the absence of magnetocrystalline anisotropy (considering only shape anisotropy). The results are shown in Figure A1.

### Appendix A.2. Dipolar Interaction

In a second stage, we consider a hexagonal array of seven conical nanostructures with the same geometric parameters as in the previous case, considering a center-to-center separation of 60 nm (see Figure A2a). Additionally, we incorporated into this array the 4.5 nm thick iron base that supports these nanostructures (see Figure A2b).

### Appendix A.3. Variation of Magnetocrystalline Anisotropy

Ultimately, we incorporate a uniaxial anisotropy in the $H_{paral}\Phi_{perp}$ direction on the isolated conical nanostructure representing the R-mask sample and study how hysteresis loops change as a function of this constant. The results obtained are presented in Figure A3.
